# The novel EGFR mutations (p.E746_S752delinsI, p.T751_I759delinsG, p.L747_S752delinsAA) in patients with non-small cell lung cancer and the clinical treatment strategy: three case reports

**DOI:** 10.3389/fonc.2023.1129629

**Published:** 2023-09-19

**Authors:** Yamin Meng, Xiaodong Li, Lei Zhang, Minhua Ye

**Affiliations:** ^1^Key Laboratory of Digital Technology in Medical Diagnostics of Zhejiang Province, Hangzhou, Zhejiang, China; ^2^Department of Cardiothoracic Surgery, Taizhou Hospital of Zhejiang Province Affiliated to Wenzhou Medical University, Linhai, Zhejiang, China

**Keywords:** NSCLC (non-small cell lung cancer), EGFR (epidermal growth factor receptor), mutation, icotinib, NGS (next-generation sequencing)

## Abstract

Epidermal growth factor receptor (*EGFR*) is an established driver gene in non-small cell lung cancer (NSCLC) and the common Exon 19 del mutation (p.E746_A750 del) has exhibited remarkable responses for EGFR tyrosine kinase inhibitors (TKIs). However, there is even less comprehension of the treatment strategy in NSCLC patients harboring uncommon Exon 19 delins mutation. Here, we identified three novel *EGFR* Exon 19 mutations (p.E746_S752delinsI, p.T751_I759delinsG, p.L747_S752delinsAA), and described the clinical treatment process. To our knowledge, the *EGFR* p.E746_S752delinsI mutation of the patient with advanced NSCLC could benefit from the treatment with Icotinib. Otherwise, for the NSCLC patients with early-stage, one harboring p.T751_I759delinsG mutation had an excellent recovery and the other harboring p.L747_S752delinsAA experienced a relapse after receiving horacoscopic radical resection, which means the patients with different Exon 19 delins mutation might have different prognosis. Our study also demonstrated that next-generation sequencing (NGS) is a crucial tool in guiding clinical treatment decisions in NSCLC. Furthermore, the real incidence of these mutation is not known, the routinely use of NGS surely will increase the detection of EGFR del-ins respect to the old tools used to screen for EGFR mutations.

## 1 Introduction

The clinical treatment of non-small cell lung cancer (NSCLC) with common *EGFR* mutations (exon 19 deletion and exon 21 L858R), has made a great breakthrough in these years, especially for targeted EGFR tyrosine kinase inhibitors (TKIs) (1–3). *EGFR* Exon 20 insertion accounts for 4%-6% of *EGFR* mutation and was almost insensitive to all three generation TKIs, which responded well to EGFR/MET bispecific antibody amivantamab (4). The generally defined “common mutation” of *EGFR* Exon 19 del refers to the short frame deletion between E746 and A750, which accounts for about 75% of *EGFR* 19 del mutations and responds well to EGFR-TKIs. However, many uncommon mutation subtypes of Exon 19 have varied responses to EGFR-TKIs, and most patients with uncommon Exon 19 mutations showed poor prognoses (5).

It is reported that patients with uncommon *EGFR* mutations show heterogeneous and reduced responses to the third-generation EGFR osimertinib (6). A multicenter, phase II trial reported that osimertinib demonstrated favorable activity in patients with a part uncommon *EGFR* mutations (7, 8). Based on the retrospective study, uncommon *EGFR* mutations including S768I, L861Q and G719X responded well to the second-generation afatinib, which was approved by US Food and Drug Administration (FDA) (9–11). Icotinib is the first-generation TKIs for NSCLC patients with *EGFR* mutation approved by China Food and Drug Administration (CFDA). However, due to tumor heterogeneity and complicated regulatory mechanism, the patients harboring uncommon *EGFR* mutation showed different susceptibility to icotinib (12–14). For example, Zhou et al. reported that an advanced LDAC patient with rare G719A/L833V double mutation of *EGFR* responded well to Icotinib and the progression-free survival was 8 months (15). While Ou et al. reported that the patient with G719D/L861Q mutations experienced progressive disease during Icotinib therapy (16). Even so, there is rare evidence reported on the efficacy of Icotinib in the uncommon *EGFR* 19 del mutation.

For these reasons, more evidence should be provided for the clinical characteristics in patients with uncommon *EGFR* 19 del mutations. Here, we identified three novel *EGFR* Exon 19 mutations and we described the clinical treatment process. Among them, a patient with advanced adenocarcinoma and EGFR p.E746_S752delinsI mutation responded well to the first-line treatment with Icotinib treatment. The other two patients harboring p.T751_I759delinsG and p.L747_S752delinsAA respectively have different prognoses.

## 2 Case presentation

The clinical characteristics of the three patients were summarized in **Table 1**.

**Table 1:** Clinical characteristics of the three patients.

Characteristics	Patient 1	Patient 2	Patient 3
**Age**	56	53	72
**Gender**	Male	Female	Male
**Tumor sizes**	1.8 cm × 2.2 cm	2.0 cm × 1.5 cm	2.0×3.0 cm
**Tumor(largest) location**	the lower lobe of the left lung	the upper lobe of the left lung	the lower lobe of the left lung
**Variant Allele Frequency**	29.62%	4.68%	19.6%
**Nucleotides mutation**	c.2235_2255 delinsAAT	c.2252_2277 delinsGG	c.2239_2254 delinsGCAG
**Amino acid mutation**	p.Glu746_Ser752 delinsIle	p.Thr751_Ile759 delinsGly	p.Leu747_Ser 752delinsAlaAla
**Therapy strategy**	Targeted therapy:Icotinib	left upper lobectomy and systematic lymph node dissection	left lower lobectomy and systematic lymph node dissection
**Diagnosis**	Invasive adenocarcinoma	Invasive adenocarcinoma	Invasive adenocarcinoma
**TNM classification**	T4N1M1b	T1N0M0	T1N0M0
**Stage of tumor**	IVA	IA	IA

### 2.1 Patient 1

**Figure 1:**
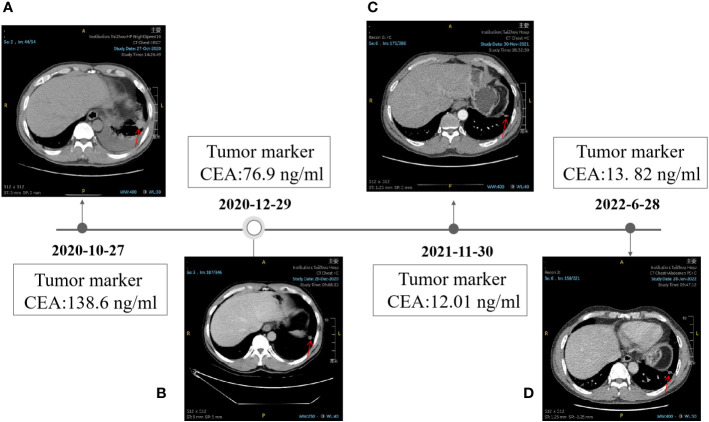
Timeline of the clinical therapy with Icotinib. **(A)** HRCT image of the patient at baseline. **(B)** HRCT image of the patient after taking Icotinib for 1.5 months, 12 months **(C)**, and 19 months **(D)**. the level of CEA was shown in the black box. The position of the tumor was denoted by red arrows.

**Figure 2:**
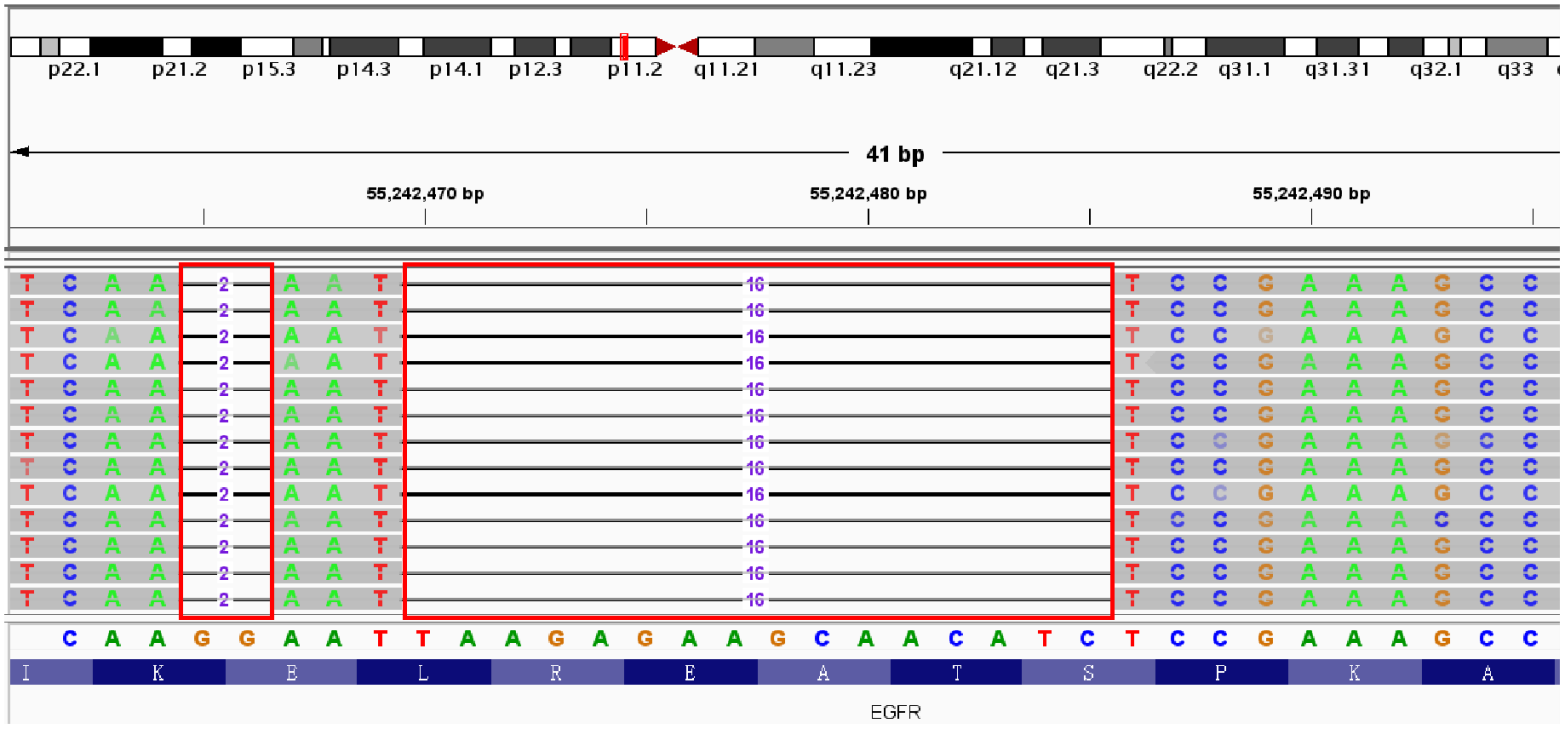
E746_S752delinsI mutation in EGFR Exon 19 identified by NGS. Red boxes: missing bases of EGFR.

A 56-year-old male patient presented to Taizhou Hospital due to paroxysmal chest pain, left pleural effusion, and bilateral pulmonary nodules on December 21, 2020. The tissue sample was acquired from a “CT-guided core biopsy of inferior lobe of left lung occupying lesion” and was sequentially sent to the Department of Pathology for a pathologic exam. High-resolution computed tomography (HRCT) scanning showed multiple nodules (1.0 cm in diameter) throughout the lung and a space-occupying lesion (1.8 cm × 2.2 cm) on the lower lobe of the left lung (**Figure 1A**). From the above results, this case was clinically diagnosed as lung adenocarcinoma (LADC) with stage IVa (T4N1M1b). A NGS panel (Dian diagnostics, Hangzhou, China), targeting ten driver mutational genes (*EGFR, KRAS, BRAF, ERBB2, NRAS, ALK, MET, RET, ROS1, PIK3CA*), highlighted an *EGFR* p.E746_S752delinsI mutation with 29.62% of Variant Allele Frequency (VAF) of the tissue sample (**Figure 2**).

The patient began Icotinib (125mg/day) on November 18, 2020, and the treatment was well tolerated with grade II stomach pain (2020.12.08-2021.7.27), grade I skin rash (2020.12.29-2021.6.15), grade I liver dysfunction (2021.02.09-2021.06.15), grade II decreasing of the lymphocyte counts (2021.10.19-2021.11.30). No grade III or IV Adverse Event (AE) and Serious Adverse Event (SAE) were observed.

After 1.5 months of treatment, The CT images showed a partiale response to treatment, in particular, the size of most nodules around the lung reduced (1.0cm to 0.7cm in diameter), the nodules on the lower lobe of the left lung rapidly shrank down from 1.8 cm × 2.2 cm to 1.3 cm×1.1 cm, and many nodules in the lung even disappeared (**Figures 1A, B**). The total follow-up time was 19 months. Up to the last follow-up, the level of CEA decreased significantly from 138.6 ng/ml to 13.82 ng/ml (**Figures 1C, D**). All these above results indicated that Icotinib was of great efficacy in the treatment of p.E746_S752delinsI mutation. Up to now, no sign of disease progression was found.

### 2.2 Patient 2

**Figure 3:**
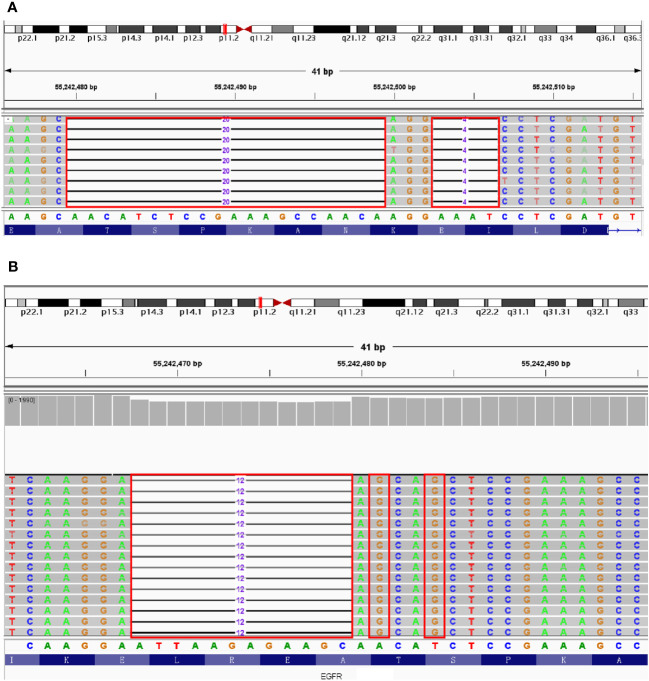
The results of NGS testing displayed by IGV. **(A)** T751_I759delinsG mutation in EGFR Exon 19. **(B)** L747_S752delinsAA mutation in EGFR Exon 19. Red boxes: missing or mutated bases of EGFR Exon19.

A 53-year-old female patient was diagnosed with ground glass nodules in the upper left lung in CT scanning during a physical examination, and therefore she presented to Taizhou Hospital for further treatment. After completing relevant examinations and excluding contraindications for surgery. The patient underwent to single-hole thoracoscopic radical resection in the left upper of the lung on May 12, 2020. During surgery, a mass about 2.0 cm × 1.5 cm in the posterior apex of the left superior lung and multiple micro-nodules in the right lung were identified. Combined with the pathological results, the patient was diagnosed with invasive stage IA adenocarcinoma (T1N0M0). Therefore, the patient was treated with left upper lobectomy and systematic lymph node dissection. The subsequent NGS testing revealed a novel *EGFR* p.T751_I759delinsG mutation with 4.68% VAF in the tumor tissue (**Figure 3A**). After 7 days, She discharged from the hospital. The total follow-up time was 24 months, and up to the last time, no sign of recurrence was found.

### 2.3 Patient 3

A 72-year-old male patient presented to Taizhou Hospital for further examination, since the abnormal CT images were found 2 days ago. The enhanced CT scan was further perfomed. a mass in the left lobe of the lung and multiple micro-nodules diffusing in the lung were identified, in the absence of abnormalities in upper abdomen. After excluding contraindications for surgery, the patient underwent a single-hole thoracoscopic radical resection of lung cancer on June 23, 2020. During surgery, 2.0×3.0 cm mass in the left lower lung and mediastinal lymphadenectasis were diagnosed. The patient was therefore treated with left lower lobectomy and systematic lymph node dissection and the pathological results confirmed the diagnosis of invasive adenocarcinoma with stage IA (T1N0M0). The NGS testing highlighted a novel *EGFR* p.L747_S752delinsAA mutation with 19.6% VAF (**Figure 3B**). After 5 days, the patient had an excellent recovery and was discharged from hospital. A first follow up visit, performed only after 1.5 years (December 7, 2021), documented the appearance of new nodules located in the contralateral lung. But unfortunately, the patient did not receive further treatment for some other reasons and was lost to follow-up.

## 3 Discussion

We identified three cases harboring novel EGFR mutations(p.E746_S752delinsI, p.T751_I759delinsG, p.L747_S752delinsAA) that were never reported before. Up to now, the advanced NSCLC patient with p.E746_S752delinsI still benefited from Icotinib treatment for a total of 19 months, with decreasing tumor burden and CEA level, which showed advanced LADC patients harboring EGFR p.E746_S752delinsI mutation could benefit from Icotinib. The other two patients of NSCLC with early-stage harboring p.T751_I759delinsG and p.L747_S752delinsAA,respectively were treated with thoracoscopic radical resection. The former had an excellent recovery and no sign of disease progression was found. However, the latter experienced a relapse after 18 months, Which means the patients with uncommon EGFR mutations have different prognoses.

In order to overcome the abnormal pathway activation and drug resistance caused by EGFR protein mutation *in vivo*, three generations of *EGFR* TKIs have been developed for the clinical treatment of NSCLC. Gefitinib and erlotinib are the representative drugs in the first-generation *EGFR* TKIs. The IPASS study illustrated that the 12-month PFS rate between the Gefitinib group and the chemotherapy group was 24.9% and 6.7%, respectively, and Gefitinib significantly reduced the risk of disease progression by 26% (17). The EVEN study indicated that the 2-years DFS rate for erlotinib versus chemotherapy was 81.35% versus 44.62% (18). The second-generation TKI, like Afatinib and Dacomitinib, were irreversible *EGFR* TKIs. LUX-Lung7 and ARCHER1050 studies established the efficacy and safety of second-generation TKI (afatiyou or dactinib) in patients with untreated EGFR-positive NSCLC (19, 20).The third-generation TKI osimertinib effectively overcome the problem of drug resistance caused by T790M mutation. The ADAURA trial, a multicenter, phase III, double-blind clinical study, found that Osimertinib reduced risk of disease recurrence or death by 83% in patients with stage II–IIIA NSCLC (21).

Patients with uncommon *EGFR* mutations show heterogeneous and reduced responses to the *EGFR* TKIs. Giuseppina Improta et al. reported the TKI sensitivity in a small cohort of lung adenocarcinomas bearing uncommon exon 19 mutations, Which described two advanced LADC patients with EGFR p.L747_T751delinsP mutation and p.T751_I759delinsS respectively who have only 2-month PFS on gefitinib therapy (22). However, an IIIB Squamous cell lung cancer (SqCLC) patient harboring EGFR Exon 19 E746_S752delinsV mutation could benefit from targeted therapies of gefitinib, and no evidence of disease was found for up to 20 months, which indicated that the first-generation EGFR TKIs might be a good choice for advanced SqCLC patients and different types of uncommon exon 19 mutations have a different response to TKIs (23). Besides, Robichaux et al. described an approach that separated the EGFR mutations into four subgroups based on structure-function and to predict the EGFR TKIs sensitivity for uncommon EGFR mutations (6).

Therefore, the above results indicate that the patients with uncommon EGFR Exon 19 mutation have a different prognosis, and it is necessary to further explore the mutation characteristic spectrum of patients with uncommon EGFR mutation and accumulate more information on prognosis. Our description of the clinical therapy process of the patients with uncommon EGFR mutations could provide a better understanding and crucial clinical guidance for personalized treatment of NSCLC patients. Importantly, the real incidence of these mutation is not known because the routinely use of NGS surely will increase the detection of EGFR del-ins respect to the old tools used to screen for EGFR mutations. However, our study has some limitations: (a) we only descripted few cases with short follow up and only one case treated with an EGFR-TKI (icotinib). Whether Icotinib is effective for all advanced NSCLC patients harboring EGFR p.E746_S752delinsI needs further confirmation. (b) The regulation mechanism of Icotinib responding well to this type of mutation is still unclear, Prospective cohort studies and experimental research are warranted for the next program. (c) Owing to various reasons, one patient with EGFR L747_S752delinsAA mutation was not receiving targeted drug therapy after relapse and his prognosis was not being tracked promptly on time.

## 4 Conclusion

Our results suggested that: (a) the patients harboring uncommon EGFR Exon 19 delins mutations have different prognoses; (b) patients with advanced LADC and EGFR p.E746_S752delinsI may benefit from the treatment of Icotinib; (c) NGS testing is crucial for guiding clinical treatment decisions in NSCLC.

## Data availability statement

The original contributions presented in the study are included in the article/supplementary material. Further inquiries can be directed to the corresponding author.

## Ethics statement

The studies involving humans were approved by Taizhou Hospital of Zhejiang Province Affiliated to Wenzhou Medical University Ethics Committee. The studies were conducted in accordance with the local legislation and institutional requirements. Written informed consent for participation was not required from the participants or the participants’ legal guardians/next of kin in accordance with the national legislation and institutional requirements. Written informed consent was obtained from the individual(s) for the publication of any potentially identifiable images or data included in this article.

## Author contributions

YM and XL were in charge of the data collection and analysis. YM and LZ were in charge of manuscript writing. MY was in charge of the manuscript revising. All authors contributed to the article and approved the submitted version.
